# Association Between Frailty and Leptin Levels in Patients with Stable Coronary Artery Disease

**DOI:** 10.3390/diagnostics16020255

**Published:** 2026-01-13

**Authors:** Kristina Krivoshapova, Daria Tsygankova, Anastasia Neeshpapa, Anastasia Kareeva, Alexander Kokov, Evgeny Bazdyrev, Victoria Karetnikova, Olga Barbarash

**Affiliations:** Research Institute for Complex Issues of Cardiovascular Diseases, 650002 Kemerovo, Russia

**Keywords:** prefrailty, frailty, coronary artery disease, percutaneous coronary intervention, leptin

## Abstract

**Background/Objectives**: The study aimed to examine the association between the total SPPB score and serum leptin levels in patients with coronary artery disease (CAD) undergoing elective percutaneous coronary intervention (PCI). **Methods**: A cross-sectional study included 204 prospectively enrolled patients with CAD who were admitted for elective PCI. The mean age was 67.45 ± 8.63 years; 63.2% of patients were male. The Short Physical Performance Battery (SPPB) was used to screen for prefrailty and frailty (10–12 points: no frailty; 8–9 points: prefrailty; ≤7 points: frailty). The levels of leptin, a biomarker of fat remodeling, were measured by a highly sensitive and highly specific enzyme immunoassay using a Diagnostics Biochem Canada Inc. Leptin ELISA Kit (London, ON, Canada). **Results**: The prevalence of frailty and prefrailty in patients with stable CAD was 20.1% and 40.2%, respectively. A comparative analysis revealed that frailty was significantly more likely in older women with CAD before elective PCI. The total serum leptin level was 13.00 [8.00–50.00] ng/mL. Frail patients with CAD had higher leptin levels than patients without frailty (25.40 [7.00–60.00] ng/mL vs. 12.00 [5.15–19.70] ng/mL, *p* = 0.037). The leptin level in patients with prefrailty was 16.70 [13.00–49.10] ng/mL. Moreover, there was a moderate inverse correlation between the total SPPB score and serum leptin levels before PCI (*p* = 0.006). A regression analysis found that the total SPPB score in patients with stable CAD was associated with high serum leptin levels (*p* < 0.001) and older age (*p* = 0.017). **Conclusions**: Our study found that frail patients with CAD undergoing PCI had higher serum leptin levels than patients without frailty.

## 1. Introduction

Frailty is one of the most common geriatric syndromes, and its prevalence is anticipated to increase in the next decades, given the progressive population aging. In frailty, even minor stress factors are associated with increased risk of falls, hospitalization, disability, and fatal outcome [1,2]. Frailty is caused by disruption of several physiological systems, including the endocrine system. Obesity, both general and abdominal, is a risk factor for pathological aging of the body [3,4]. Adipokines, adipocyte-secreted proteins, may modulate the effect of obesity on cardiovascular risk and mortality [5,6]. Leptin, the first discovered adipokine hormone, is crucial for neuroendocrine function and metabolism, as well as inflammation, immunity, angiogenesis, vascular function, bone homeostasis, and reproductive function [7,8]. In healthy individuals, elevated serum leptin levels promote satiety and energy expenditure while reducing food intake [9,10]. Leptin levels increase in obesity and are generally higher in women than in men [11]. Previous studies suggest that leptin may play a role in cardiovascular diseases (CVDs) by regulating blood pressure, glucose levels, insulin sensitivity, fatty acid catabolism, platelet aggregation, angiogenesis, and inflammatory vascular responses [12,13]. Nonetheless, the relationship between elevated leptin levels and the risk of CVDs is controversial. A meta-analysis of eight studies by Zeng et al. [14] found a significant association between high serum leptin levels and increased risk of CVDs. However, recent studies do not support these findings [15,16]. Leptin is known to regulate energy balance by targeting numerous central and peripheral tissues; however, its involvement in pathological aging is insufficiently studied. Several studies in older individuals revealed elevated serum leptin levels in frail patients, which may be attributable to leptin resistance [17,18].

Given the inconsistencies in the available data, we sought to assess the association between the total SPPB score and serum leptin levels in patients with coronary artery disease (CAD) before elective percutaneous coronary intervention (PCI).

## 2. Materials and Methods

A cross-sectional study included 204 prospectively enrolled patients with CAD who were admitted to the Cardiology Department of the Research Institute for Complex Issues of Cardiovascular Diseases (Kemerovo) for elective PCI between 2023 and 2024. All patients provided informed consent to participate in the study. The study protocol was approved by the Institutional Review Board of the Research Institute for Complex Issues of Cardiovascular Diseases (Minutes No. 12 of 27 December 2019). The diagnosis of CAD was confirmed based on the European Society of Cardiology guidelines for the management of chronic coronary syndromes (ESC-2019) [19], the presence of anginal chest pain or equivalent, medical history, and imaging findings, including electrocardiography (ECG), echocardiography, daily ECG monitoring, and coronary angiography. The Short Physical Performance Battery (SPPB), a 3-part performance-based test (gait speed, chair stand, and balance tests) was used to screen for prefrailty and frailty before PCI (10–12 points: no frailty; 8–9 points: prefrailty; ≤7 points: frailty). This test evaluates lower extremity functioning, which has been shown to correlate with mobility, disability, and treatment outcomes, including repeat hospitalizations and mortality [20].

Inclusion criteria were age over 60 years; stable CAD at admission (NYHA functional class (FC) I–III angina pectoris and/or postinfarction cardiosclerosis); and hospitalization for elective PCI. Exclusion criteria were neuromuscular diseases; grade IV chronic heart failure; uncontrolled hypertension; CAD with concomitant valvular heart diseases; severe comorbidities that worsen the mental and physical status; traumatic brain injuries; polymedication (oral steroids, antidepressants, barbiturates, muscle relaxants); inability to understand and/or follow the study protocol procedures; and refusal to participate (withdrawal of consent). All patients in the study received basic therapy for CAD.

Patients were advised to avoid physical and emotional stress for 24 h prior to the test. Furthermore, they were instructed to avoid fatty foods for 24 h and refrain from smoking for three hours before blood sampling. Samples for a fasting blood test were collected between 08:00 and 09:00 in a vacuum tube with a separating gel (yellow cap), strictly up to the mark on the tube label. The samples were then mixed by inverting a tube 5–6 times. Following blood sampling, the tubes with serum gel (yellow caps) were allowed to stand for 20–30 min before centrifugation at 1500–2000× *g* for 10 min, no earlier than 30 min and no later than 60 min after sampling. The gel tubes were not re-centrifuged to prevent hemolysis. Prior to collecting biospecimens, the tubes were stored vertically in a rack at +2 °C to +8 °C. The obtained blinded biospecimens were stored at –20 °C for two months.

Serum leptin levels in the study sample were assessed by a highly sensitive and highly specific enzyme immunoassay using a Diagnostics Biochem Canada Inc. Leptin ELISA Kit (London, ON, Canada). The Leptin ELISA is a two-step capture or “sandwich” type immunoassay. The absorbance is measured on a microplate reader at 450 nm. A set of standards is used to plot a standard curve from which the amount of leptin in patient samples and controls can be directly read. The detection range is 1–100 ng/mL. In a recent study, the reference interval for serum leptin was 0.33–19.85 ng/mL in healthy men and 3.60–54.86 ng/mL in healthy women [21].

Statistical analysis was performed using IBM SPSS Statistics 26.0.0 (USA). Qualitative variables were described using absolute and relative values (%). Continuous variables were presented as means and standard deviations, or as medians and 25th–75th percentiles, based on the type of data distribution. The Shapiro–Wilk test was used for normality testing. The Pearson’s chi-squared test with exact *p* values was used to assess differences in distribution between qualitative variable categories in three independent frailty groups (without frailty, prefrailty, frailty). The Mann–Whitney U test was used for intergroup comparisons by a non-normal quantitative variable. Comparisons of quantitative variable medians between three independent frailty groups by a quantitative variable were performed using the Kruskal–Wallis test with the Holm–Bonferroni correction. Logistic regression was used to assess associations between various qualitative and quantitative patient characteristics in the study sample and the presence of frailty according to the SPPB score. The presence of association was assessed using odds ratios (ORs) and 95% confidence intervals (CIs). The direction and strength of correlation between two quantitative variables were assessed using Spearman’s rank correlation coefficient (for non-normal variables). Linear regression was used to create a predictive model of the association between a quantitative variable and factors. ROC analysis was used to assess the discriminatory ability of quantitative variables in predicting pathological aging (prefrailty and frailty according to the SPPB score). The highest Youden’s index value was used to determine a quantitative variable’s cut-off value. The differences were considered significant at a two-sided *p* ≤ 0.05.

## 3. Results

The mean age was 67.45 ± 8.63 years; 63.2% of patients were male. The majority of patients had hypertension (195 patients; 95.6%), and 51.0% (104 patients) had a history of myocardial infarction. Type 2 diabetes mellitus was reported in one-third of patients (63 patients; 30.9%). The majority of patients were overweight (body mass index [BMI] 29.53 ± 5.78 kg/m^2^). Increased body weight was reported in 70 (34.3%) patients, grade I obesity in 57 (27.9%), grade II obesity in 26 (12.7%), grade III obesity in 5 (2.5%), and underweight in 6 (3.0%). Paroxysmal atrial fibrillation was observed in 37 (18.1%) patients. Coronary angiography revealed double- and triple-vessel CAD in the majority of patients. Moreover, half of the patients in the study sample had a history of revascularization, and 20 (9.8%) patients had a history of stroke. The median left ventricular ejection fraction (LVEF) according to echocardiography was 60.50 [44.25–65.75]%. Blood chemistry findings were within the normal range (see Table 1).

The total serum leptin level in the study sample was 13.00 [8.00–50.00] ng/mL (see Table 1). Notably, the median serum leptin level was several times higher in female patients with CAD before elective PCI (*p* < 0.001, see Table 2).

**Figure 1:**
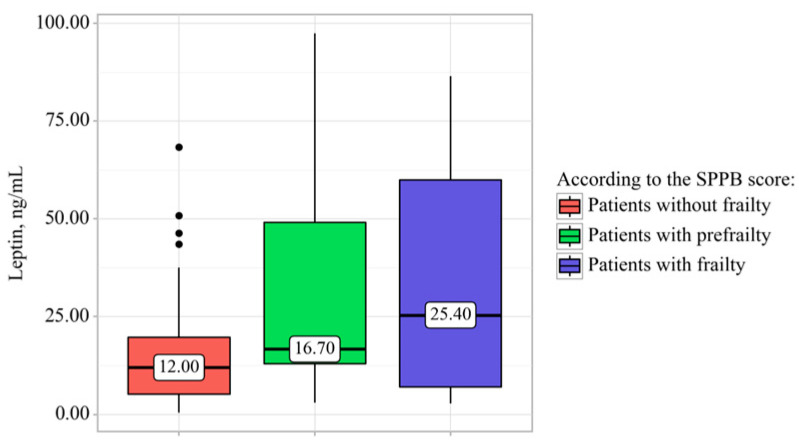
Serum leptin levels in patients with coronary artery disease, depending on the presence of pathological aging.

The prevalence of prefrailty in patients with CAD was quite high: 40.2% (82 patients), whereas frailty was detected in 20.1% of cases (41 patients) (see Table 3). Frailty was significantly more likely in older women with CAD before elective PCI. There were no other significant differences in the clinical characteristics and medical history of patients with variable manifestations of pathological aging. Serum hemoglobin levels in patients without frailty were significantly higher (136.62 ± 12.04 [95% CI, 133.95–139.30] g/L) than in patients with prefrailty (131.27 ± 14.03 [95% CI, 128.12–134.41] g/L, *p* = 0.035) and frailty (129.27 ± 14.99 [95% CI, 124.54–134.00] g/L, *p* = 0.014). Serum leptin levels were significantly higher in frail patients with CAD than in patients without frailty: 25.40 [7.00–60.00] ng/mL vs. 12.00 [5.15–19.70] ng/mL, *p* = 0.037 (see Figure 1). An additional analysis of sex-related distribution of leptin levels in each group revealed no significant differences (*p* > 0.05). There were no differences in the BMI and body weight changes between patients with varying degrees of frailty (*p* > 0.05). Furthermore, there were no significant intergroup differences in glucose, creatinine, and total cholesterol levels. Echocardiography revealed that patients with frailty had a significantly lower LVEF than patients with prefrailty (51.00 [32.00–64.00]% vs. 64.00 [61.00–70.00]%, *p* = 0.020).

Multivariate logistic regression was used to identify associations between various qualitative and quantitative patient characteristics in the study sample and the presence of frailty according to the SPPB score. Significant differences were observed only for age (*p* = 0.032) and serum leptin levels (*p* = 0.042) of patients with CAD (see Table 4).

Next, a correlation analysis was performed to assess the association between leptin levels and frailty. The analysis included testing the correlation coefficient between frailty score and each of the 8 independent variables of interest (leptin, ng/mL; age, years; BMI, kg/m^2^; LVEF, %; hemoglobin, g/L; glucose, mmol/L; creatinine, μmol/L; total cholesterol, mmol/L). The analysis revealed a moderate inverse correlation between the total SPPB score and serum leptin levels in patients with CAD before PCI (ρ = −0.318, *p* = 0.006).

**Figure 2:**
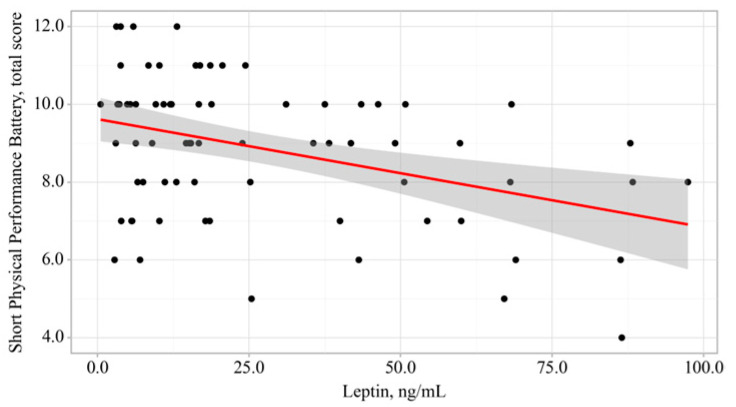
Regression chart of the association between the total Short Physical Performance Battery score and serum leptin levels in patients with coronary artery disease.

The observed relationship is described by the simple linear regression equation:

(1) Y_SPPB, total score_ = −0.028 × X_leptin, ng/mL_ + 9.619,

With a 1 ng/mL increase in the serum leptin level in patients with CAD, a 0.028 decrease in the total SPPB score can be expected; the proposed model explains 15.5% of the observed variance (see Figure 2).

**Figure 3:**
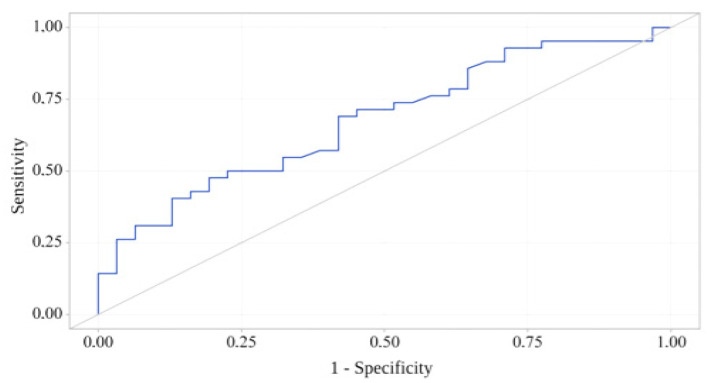
ROC curve characterizing the discriminatory ability of serum leptin levels (ng/mL) in predicting pathological aging.

A ROC analysis showed that serum leptin levels had adequate discriminatory power to discriminate between patients with and without frailty (area under the ROC curve [AUC] = 0.788; 95% CI, 0.655–0.899, *p* = 0.010). Serum leptin levels were significantly higher in frail patients with CAD than in patients without frailty according to the SPPB score: 21.20 [10.42–53.45] ng/mL vs. 12.00 [5.15–19.70] ng/mL, *p* = 0.010. The cut-off leptin level was 25.20 ng/mL, with a sensitivity and specificity of 59.6% and 72.6%, respectively (see Figure 3).

Linear regression was used to develop a prognostic model that characterizes the relationship between lower extremity functioning in patients with CAD and various quantitative factors. The final significant model (*p* < 0.001) included age and serum leptin levels of patients in the study sample (Table 5). We used variable reduction to obtain the final model (stepwise multiple regression).

The observed relationship is described by the linear regression equation:

(2) Y_SPPB, total score_ = 13.642 − 0.061X_age, years_ − 0.026X_leptin, ng/mL_,

where Y_SPPB, total score_ is the total SPPB score, X_age, years_ is age (years), and X_leptin, ng/mL_ is serum leptin level in patients with CAD (ng/mL).

With a one-year increase in age, a 0.061-point decrease in the total SPPB score can be expected, whereas an increase in the leptin level by 1 ng/mL will result in a decrease in the total SPPB score by 0.026 points. The resulting regression model has a correlation coefficient rxy = 0.470, which corresponds to a moderate correlation on the Chaddock scale. These variables explain 28.1% of the total SPPB score variability.

## 4. Discussion

Chronic subclinical pro-inflammatory status can lead to various complex immune and hormonal changes in older individuals. According to some researchers, pro-inflammatory myokines, particularly leptin, have a negative effect on muscle mass and strength, which can promote frailty [22]. Leptin, a fatty cytokine primarily secreted by white adipose tissue, participates in a variety of pathophysiological processes, such as maintaining energy balance and regulating immune and inflammatory responses, as well as bone and muscle metabolism [23]. However, there are very few studies confirming the critical role of leptin in pathological aging. Some researchers highlight the negative effects of high serum leptin levels on the cardiovascular system and aging of the body, while others consider leptin as a neutral, and in some cases even a protective factor.

Our findings indicate that the prevalence of both prefrailty and frailty in patients with CAD is quite high, especially in older patients (prefrailty: 82 patients, 40.2%; frailty: 41 patients, 20.1%). Moreover, frail patients had higher serum leptin levels than patients without frailty (25.40 [7.00–60.00] ng/mL, *p* = 0.037), with a moderate inverse correlation between the total SPPB score and serum leptin levels before PCI (*p* = 0.006). The regression analysis revealed an association between the total SPPB score in patients with stable CAD and high serum leptin levels (*p* < 0.001), as well as older age (*p* = 0.017). These findings may be associated with reduced sympathetic nervous activity, resulting in high plasma leptin levels in frail older adults. High leptin levels promote inflammatory cytokine-induced muscle atrophy. Sympathetic nerve fibers innervate adipose tissue and mediate the lipolytic effect of leptin; therefore, elevated plasma leptin levels and reduced sympathetic nervous activity may contribute to muscle wasting. Notably, patients with the most severe functional impairments in the study sample naturally belonged to the cohort of sensitive patients, which could be one of the key factors underlying the high serum leptin level as a marker of multisystem changes.

In a Chinese study in patients with emaciation over 65 years of age, leptin was one of the factors associated with frailty (*p* < 0.05) [17]. A relationship has been reported between high serum leptin levels and chronic inflammation in frail patients, as subclinical inflammation results in elevated leptin levels. Furthermore, it has been hypothesized that leptin sensitivity may decrease with age due to impaired hypothalamic leptin receptor signaling [24]. Martín et al. [25] found that leptin impairs the prognosis in cancers and neurodegenerative disorders by increasing cell susceptibility to inflammatory mediators, supporting its potential as a biomarker and therapeutic target in pathological aging studies. In animal studies, serum leptin levels increased significantly with age [26]. In contrast, Hubbard et al. [27] found that frail older patients with cachexia had reduced serum leptin levels. These findings may be associated with the course of frailty. The role of leptin in the pathogenesis of atherosclerosis also remains uncertain. A large meta-analysis of 13 epidemiological studies in 4257 patients with CVD and 26,710 controls found no association between elevated serum leptin levels and the risk of CAD or stroke [28]. Other studies indicate a link between hyperleptinemia and several CVDs, including congestive heart failure, myocardial infarction, and CAD [29,30,31]. Thus, the association between high serum leptin levels and increased pathogenetic risk of CAD or pathological aging remains unclear. Nevertheless, leptin can become one of the key factors in the synergy of atherosclerosis and frailty, considerably increasing mortality [32]. Moreover, leptin is a promising therapeutic target for preventing CVDs and pathological aging. Notably, emerging comparative data in frail patients with type 2 diabetes mellitus suggest therapy class-associated differences in cardiovascular and glycemic endpoints, underscoring the need to interpret biomarker–frailty associations within an evolving therapeutic context [33].

Our study had several limitations. Most variables, including leptin levels and the total SPPB score, were assessed using standardized, validated methods; however, the study did not include a healthy control group. Furthermore, there were no reference values for serum leptin levels, and the study did not include leptin resistance assessment. The AUC of 0.788 reported in our study indicates moderate discriminatory ability. Moreover, the selected cut-off value of 25.2 ng/mL results in only 59.6% sensitivity and 72.6% specificity, which is at best mediocre classification performance and may not be robust, especially in a single-center sample without internal (bootstrap/cross-validation) or external validation. Nevertheless, the findings have substantial scientific promise, considering that the study examined an extremely vulnerable category of patients with CAD.

## 5. Conclusions

Our findings demonstrate an association between the total SPPB score and high serum leptin levels in older patients with stable CAD. However, further research is needed to study the role of leptin in pathological aging in various populations of older patients.

## Figures and Tables

**Table 1:** Clinical and laboratory characteristics of patients with stable coronary artery disease.

Parameters	Patient Characteristics (n = 204)
Mean age, years, M ± SD	67.45 ± 8.63
Male, n (%)	129 (63.2)
BMI, kg/m^2^, M ± SD	29.53 ± 5.78
Normal body weight, n (%)	40 (19.6)
Overweight, n (%)	70 (34.3)
Grade I obesity, n (%)	57 (27.9)
Grade II obesity, n (%)	26 (12.7)
Grade III obesity, n (%)	5 (2.5)
Underweight, n (%)	6 (3.0)
Smokers/quitters for <3 months, n (%)	58 (28.4)
PICS, n (%)	104 (51.0)
FC II–III CHF, n (%)	182 (89.2)
LVEF, %, Me [Q1–Q3]	60.50 [44.25–65.75]
History of PCI, n (%)	102 (50.0)
Single-vessel CAD, n (%)	45 (22.0)
Double-vessel CAD, n (%)	75 (36.8)
Triple-vessel CAD, n (%)	84 (41.2)
History of stroke, n (%)	20 (9.8)
Hypertension, n (%)	195 (95.6)
Preoperative AF, n (%)	37 (18.1)
Type 2 DM, n (%)	63 (30.9)
COPD, n (%)	9 (4.4)
Hemoglobin, g/L, M ± SD	132.36 ± 14.02
Glucose, mmol/L, Me [Q1–Q3]	5.70 [5.20–6.50]
Creatinine, µmol/L, Me [Q1–Q3]	87.00 [76.00–100.75]
Total cholesterol, mmol/L, Me [Q1–Q3]	4.00 [3.50–4.90]
Leptin, ng/mL, Me [Q1–Q3]	13.00 [8.00–50.00]

Note: AF, atrial fibrillation; BMI, body mass index; CAD, coronary artery disease; CHF, chronic heart failure; COPD, chronic obstructive pulmonary disease; DM, diabetes mellitus; FC, functional class; LVEF, left ventricular ejection fraction; PCI, percutaneous coronary intervention; PICS, postinfarction cardiosclerosis.

**Table 2:** Serum leptin levels in patients with coronary artery disease depending on sex.

Categories	Leptin, ng/mL, Me [Q1–Q3]	*p*-Value
Male, n = 129 (63.2%)	10.55 [5.42–18.52]	<0.001 *
Female, n = 75 (36.8%)	35.60 [15.65–59.90]	

Note: *, significant differences, *p* ≤ 0.05.

**Table 3:** Clinical and laboratory characteristics of patients before elective percutaneous coronary intervention, depending on the presence of prefrailty and frailty.

Characteristics	Patients Without Frailty, n_0_ = 81 (39.7%)	Patients with Prefrailty, n_1_ = 82 (40.2%)	Patients with Frailty, n_2_ = 41 (20.1%)	*p*-Value
Mean age, years, M ± SD (95% CI)	64.85 ± 9.09 (62.84–66.86)	67.29 ± 8.30 (65.47–69.12)	71.00 ± 7.29 (68.70–73.30)	<0.001 * p_w/o F–F_ <0.001 *
Male, n (%)	62 (76.5)	47 (57.3)	20 (48.8)	0.004 * *p*_w/o F–PF_ = 0.018 * *p*_w/o F–F_ = 0.006 *
Female, n (%)	19 (23.5)	35 (42.7)	21 (51.2)	
BMI, kg/m^2^, M ± SD (95% CI)	28.60 ± 4.72 (27.55–29.64)	29.82 ± 5.41 (28.63–31.01)	30.72 ± 7.01 (28.51–32.93)	0.111
Normal body weight, n (%)	19 (23.5)	13 (15.9)	8 (19.5)	0.658
Overweight, n (%)	22 (27.2)	33 (40.2)	15 (36.6)	
Grade I obesity, n (%)	25 (30.9)	22 (26.8)	10 (24.4)	
Grade II obesity, n (%)	12 (14.8)	8 (9.8)	6 (14.6)	
Grade III obesity, n (%)	2 (2.5)	3 (3.7)	0 (0.0)	
Underweight, n (%)	1 (1.2)	3 (3.7)	2 (4.9)	
Smokers/quitters for <3 months, n (%)	20 (24.7)	28 (34.1)	10 (24.4)	0.333
PICS, n (%)	41 (50.6)	38 (46.3)	25 (61.0)	0.309
FC II–III CHF, n (%)	74 (91.4)	71 (86.6)	37 (90.3)	0.858
LVEF, %, Me [Q1–Q3]	54.50 [43.50–61.25]	64.00 [61.00–70.00]	51.00 [32.00–64.00]	0.003 * *p*_PF–w/o F_ = 0.005 * *p*_F–PF_ = 0.020 *
History of PCI, n (%)	41 (50.6)	36 (43.9)	25 (61.0)	0.201
Single-vessel CAD, n (%)	24 (29.6)	16 (19.6)	5 (12.2)	0.181
Double-vessel CAD, n (%)	23 (28.4)	33 (40.2)	19 (46.3)	
Triple-vessel CAD, n (%)	34 (42.0)	33 (40.2)	17 (41.5)	
History of stroke, n (%)	6 (7.4)	9 (11.0)	5 (12.2)	0.632
Hypertension, n (%)	76 (93.8)	80 (97.6)	39 (95.1)	0.503
Preoperative AF, n (%)	15 (18.5)	18 (22.0)	4 (9.8)	0.253
Type 2 DM, n (%)	25 (30.9)	29 (35.4)	9 (22.0)	0.316
COPD, n (%)	5 (6.2)	3 (3.7)	1 (2.4)	0.581
Hemoglobin, g/L, M ± SD (95% CI)	136.62 ± 12.04 (133.95–139.30)	131.27 ± 14.03 (128.12–134.41)	129.27 ± 14.99 (124.54–134.00)	0.007 * *p*_w/o F–PF_ = 0.035 * *p*_w/o F–F_ = 0.014 *
Glucose, mmol/L, Me [Q1–Q3]	5.60 [5.10–6.22]	5.80 [5.45–6.70]	5.80 [5.40–7.40]	0.074
Creatinine, μmol/L, Me [Q1–Q3]	90.00 [76.50–105.50]	89.00 [78.00–99.50]	85.00 [75.00–96.00]	0.520
Total cholesterol, mmol/L, Me [Q1–Q3]	4.00 [3.50–5.00]	3.90 [3.42–4.97]	3.90 [3.50–4.75]	0.640
Leptin, ng/mL, Me [Q1–Q3]	12.00 [5.15–19.70]	16.70 [13.00–49.10]	25.40 [7.00–60.00]	0.037 *

Note: AF, atrial fibrillation; BMI, body mass index; CAD, coronary artery disease; CHF, chronic heart failure; COPD, chronic obstructive pulmonary disease; DM, diabetes mellitus; F, frailty; FC, functional class; LVEF, left ventricular ejection fraction; PCI, percutaneous coronary intervention; PF, prefrailty; PICS, postinfarction cardiosclerosis; w/o F, patients without frailty; PF, patients with prefrailty; F, patients with frailty; *, significant differences, *p* ≤ 0.05.

**Table 4:** Results of the multivariate logistic model to assess the correlates of frailty.

Variables	Presence of Frailty			
	Odds Ratio	95% Confidence Interval		*p*-Value
Age, years	1.277	1.120	1.598	0.032 *
Sex	1.364	0.230	8.085	0.733
BMI, kg/m^2^	1.039	0.918	1.176	0.550
Type 2 DM	0.944	0.144	6.190	0.952
Preoperative AF	1.048	0.193	5.692	0.862
PICS	0.444	0.106	1.866	0.268
LVEF, %	1.001	0.967	1.038	0.942
Leptin, ng/mL	1.432	1.200	1.665	0.042 *
Creatinine, μmol/L	1.017	0.983	1.051	0.334

Note: AF, atrial fibrillation; BMI, body mass index; DM, diabetes mellitus; LVEF, left ventricular ejection fraction; PICS, postinfarction cardiosclerosis; *, significant differences, *p* ≤ 0.05.

**Table 5:** Regression for the dependent variable: total Short Physical Performance Battery score.

Predictors	B	Standard Error	*t*	*p*-Value
Intercept	13.642	1.673	8.155	<0.001 *
Age, years	−0.061	0.025	−2.438	0.017 *
Leptin, ng/mL	−0.026	0.007	−3.527	<0.001 *

Note: *, significant differences, *p* ≤ 0.05.

## Data Availability

The original contributions presented in this study are included in the article. Further inquiries can be directed to the corresponding author.

## Abbreviations

AF	Atrial fibrillation
BMI	Body mass index
CAD	Coronary artery disease
CHF	Chronic heart failure
CI	Confidence interval
COPD	Chronic obstructive pulmonary disease
CVD	Cardiovascular disease
DM	Diabetes mellitus
FC	Functional class
LVEF	Left ventricular ejection fraction
PCI	Percutaneous coronary intervention
PICS	Postinfarction cardiosclerosis
SPPB	Short Physical Performance Battery
